# What feedback do reviewers give when reviewing qualitative manuscripts? A focused mapping review and synthesis

**DOI:** 10.1186/s12874-020-01005-y

**Published:** 2020-05-18

**Authors:** Oliver Rudolf HERBER, Caroline BRADBURY-JONES, Susanna BÖLING, Sarah COMBES, Julian HIRT, Yvonne KOOP, Ragnhild NYHAGEN, Jessica D. VELDHUIZEN, Julie TAYLOR

**Affiliations:** 1 Institute of General Practice, Centre for Health and Society, Medical Faculty of the Heinrich Heine University Düsseldorf, Moorenstr. 5, 40225 Düsseldorf, Germany; 2 School of Nursing, College of Medical and Dental Sciences, University of Birmingham, Birmingham, UK; 3 Institute of Health and Care Sciences, Sahlgrenska Academy at the University of Gothenburg, Gothenburg, Sweden; 4 Florence Nightingale Faculty of Nursing, Midwifery & Palliative Care, King’s College London, London, UK; 5 Institute of Applied Nursing Sciences, Department of Health, University of Applied Sciences FHS St.Gallen, St. Gallen, Switzerland; 6 Cardiology department, Radboud University Medical Centre, Nijmegen, the Netherlands; 7 Division of Emergencies and Critical Care, Oslo University Hospital/Institute of Health and Society, Faculty of Medicine, University of Oslo, Oslo, Norway; 8 Hogeschool Utrecht, Utrecht, the Netherlands; 9 Birmingham Women’s and Children’s Hospitals NHS Foundation Trust, Birmingham, UK

**Keywords:** Open access publishing, Journals, Peer review, Manuscript review, reviewer’s report, Qualitative analysis, Qualitative research, Synthesis, Mapping

## Abstract

**Background:**

Peer review is at the heart of the scientific process. With the advent of digitisation, journals started to offer electronic articles or publishing online only. A new philosophy regarding the peer review process found its way into academia: the open peer review. Open peer review as practiced by BioMed Central (*BMC*) is a type of peer review where the names of authors and reviewers are disclosed and reviewer comments are published alongside the article. A number of articles have been published to assess peer reviews using quantitative research. However, no studies exist that used qualitative methods to analyse the content of reviewers’ comments.

**Methods:**

A focused mapping review and synthesis (FMRS) was undertaken of manuscripts reporting qualitative research submitted to *BMC* open access journals from 1 January – 31 March 2018. Free-text reviewer comments were extracted from peer review reports using a 77-item classification system organised according to three key dimensions that represented common themes and sub-themes. A two stage analysis process was employed. First, frequency counts were undertaken that allowed revealing patterns across themes/sub-themes. Second, thematic analysis was conducted on selected themes of the narrative portion of reviewer reports.

**Results:**

A total of 107 manuscripts submitted to nine open-access journals were included in the FMRS. The frequency analysis revealed that among the 30 most frequently employed themes “writing criteria” (dimension II) is the top ranking theme, followed by comments in relation to the “methods” (dimension I). Besides that, some results suggest an underlying quantitative mindset of reviewers. Results are compared and contrasted in relation to established reporting guidelines for qualitative research to inform reviewers and authors of frequent feedback offered to enhance the quality of manuscripts.

**Conclusions:**

This FMRS has highlighted some important issues that hold lessons for authors, reviewers and editors. We suggest modifying the current reporting guidelines by including a further item called “Degree of data transformation” to prompt authors and reviewers to make a judgment about the appropriateness of the degree of data transformation in relation to the chosen analysis method. Besides, we suggest that completion of a reporting checklist on submission becomes a requirement.

## Background

Peer review is at the heart of the scientific process. Reviewers independently examine a submitted manuscript and then recommend acceptance, rejection or – most frequently – revisions to be made before it gets published [1]. Editors rely on peer review to make decisions on which submissions warrant publication and to enhance quality standards. Typically, each manuscript is reviewed by two or three reviewers [2] who are chosen for their knowledge and expertise regarding the subject or methodology [3]. The history of peer review, often regarded as a “touchstone of modern evaluation of scientific quality” [4] is relatively short. For example, the *British Medical Journal* (now the *BMJ*) was a pioneer when it established a system of external reviewers in 1893. But it was in the second half of the twentieth century that employing peers as reviewers became custom [5]. Then, in 1973 the prestigious scientific weekly *Nature* introduced a rigorous formal peer review system for every paper it printed [6].

Despite ever-growing concerns about its effectiveness, fairness and reliability [4, 7], peer review as a central part of academic self-regulation is still considered the best available practice [8]. With the advent of digitisation in the late 1990s, scholarly publishing has changed dramatically with many journals starting to offer print as well as electronic articles or publishing online only [9]. The latter category includes for-profit journals such as BioMed Central (*BMC*) that have been online since their inception in 1999, with an ever evolving portfolio of currently over 300 peer-reviewed journals.

As compared to traditional print journals where individuals or libraries need to pay a fee for an annual subscription or for reading a specific article, open access journals such as BMC, PLoS ONE or BMJ Open are permanently free for everyone to read and download since the cost of publishing is paid by the author or an entity such as the university. Many, but not all, open access journals impose an article processing charge on the author, also known as the gold open access route, to cover the cost of publication. Depending on the journal and the publisher, article processing charges can range significantly between US$100 and US$5200 per article [10, 11].

In the digital age, a new philosophy regarding the peer review process found its way into academia, questioning the anonymity of the closed system of peer-review as contrary to the demands for transparency [1]. The issue of reviewer bias, especially concerning gender and affiliation [12], led not only to the establishment of double-blind review but also to its extreme opposite: the open peer review system [8]. Although the term ‘open peer review’ has no standardised definition, scholars use the term to indicate that the identities of the authors and reviewers are disclosed and that reviewer reports are openly available [13]. In the late 1990s, the *BMJ* changed from a closed system of peer review to an open system [14, 15]. During the same time, other publishers such as some journals in *BMC* followed the example of opening up their peer review.

While peer review reports have long been hidden from the public gaze [16, 17], opening up the closed peer review system allows researchers to access reviewer comments, thus making it possible to study them. Since then, a number of articles have been published to assess reviews using quantitative research methods. For example, Landkroon et al. [18] assessed the quality of 247 reviews of 119 original articles using a 5-point Likert scale. Similarly, Henly and Dougherty [19] developed and applied a grading scale to assess the narrative portion of 464 reviews of 203 manuscripts using descriptive statistics. The retrospective cohort study by van Lent et al. [20] assessed peer review comments on drug trials from 246 manuscripts to investigate whether there is a relationship between the content of these comments and sponsorship using a generalised linear mixed model. Most recently, Davis et al. [21] evaluated reviewer grading forms for surgical journals with higher impact factors and compared them to surgical journals with lower impact factors using Fisher’s exact test.

Despite the readily available reviewer comments that are published alongside the final article of many open access journals, to the best of our knowledge no studies exist to date that used – besides quantitative methods – also qualitative methods to analyse the content of reviewers’ comments. Identifying (negative) reviewer comments will help authors to pay particular attention to these aspects and assist prospective qualitative researchers to understand the most common pitfalls when preparing their manuscript for submission. Thus, the aim of the study was to appraise the quality and nature of reviewers’ feedback in order to understand how reviewers engage with and influence the development of a qualitative manuscript. Our focus on qualitative research can be explained by the fact that we are passionate qualitative researchers with a history in determining the state of qualitative research in health and social science literature [22]. The following research questions were answered: (1) What are the frequencies of certain commentary types in manuscripts reporting on qualitative research? and (2) What are the nature of reviewers’ comments made on manuscripts reporting on qualitative research?

## Methods

We conducted a focused mapping review and synthesis (FMRS) [22–25]. Most forms of review aim for breadth and exhaustive searches, but the FMRS searches within specific, pre-determined journals. While Platt [26] observed that ‘a number of studies have used samples of journal articles’, the distinctive feature of the FMRS is the purposive selection of journals. These are chosen for their likelihood to contain articles relevant to the field of inquiry – in this case qualitative research published in open access journals that operate an open peer-review process that involves posting the reviewer’s reports. It is these reports that we have analysed using thematic analysis techniques [27].

Currently there are over 70 BMC journals that have adopted open peer-review. The FMRS focused on reviewers’ reports published during the first quarter of 2018. Journals were selected using a three-stage process. First, we produced a list with all BMC journals that operate an open peer review process *and* will publish qualitative research articles (*n* = 62). Second, from this list we selected journals that are general fields of practice and non-disease specific (*n* = 15). Third, to ensure a sufficient number of qualitative articles, we excluded journals with less than 25 hits on the search term “qualitative” for the year 2018 (search date: 16 July 2018) because chances were considered too slim to contain sufficient articles of interest. At the end of the selection process, the following nine BMC journals were included in our synthesis: (1) *BMC Complementary and Alternative Medicine*, (2) *BMC Family Practice*, (3) *BMC Health Services Research*, (4) *BMC Medical Education*, (5) *BMC Medical Ethics*, (6) *BMC Nursing*, (7) *BMC Public Health*, (8) *Health Research Policy and Systems*, and (9) *Implementation Science*. Since these journals represent different subjects, a variety of qualitative papers written for different audiences was captured. Every article published within the timeframe was scrutinised against the inclusion and exclusion criteria (Table 1).

**Table 1:** Inclusion and exclusion criteria

Inclusion criteria	Exclusion criteria
Primary empirical studies	Systematic reviews
Qualitative research	Quantitative studies
	Mixed method studies
Published between 1st January & 31st March 2018	Secondary data analysis
	Methodological & theoretical articles

### Development of the data extraction sheet

A validated instrument for the classification of reviewer comments does not exist [20]. Hence, a detailed classification system was developed and pilot tested considering previous research [20]. Our newly developed data extraction sheet consists of a 77-item classification system organised according to three dimensions: (1) scientific/technical content, (2) writing criteria/representation, and (3) technical criteria. It represents themes and sub-themes identified by reading reviewer comments from twelve articles published in open peer-review journals. For the development of the data extraction sheet, we randomly selected four articles containing qualitative research from each of the following three journals published between 2017 and 2018: *BMC Nursing*, *BMC Family Practice* and *BMJ Open*. We then analysed the reviews of manuscripts by systematically coding and categorising the reviewers’ free-text comments. Following the recommendation by Shashok [28], we initially organised the reviewer’s comments along two main dimensions, i.e., scientific content and writing criteria. Shashok [28] argues that when peer reviewers confuse content and writing, their feedback can be misunderstood by authors who may modify texts in unintentional ways to the detriment of the manuscript.

To check the comprehensiveness of our classification system, provisional themes and sub-themes were piloted using reviewer comments we had previously received from twelve of our own manuscripts that had been submitted to journals that operate blind peer-review. We wanted to account for potential differences in reviewers’ feedback (open vs. blind review). As a result of this quality enhancement procedure, three sub-themes and a further dimension (‘technical criteria’) were added. For reasons of clarity and comprehensibility, the dimension ‘scientific content’ was subdivided following the IMRaD structure. IMRaD is the most common organisational structure of an original research article comprising **I**ntroduction, **M**ethods, **R**esults **a**nd **D**iscussion [29]. Anchoring examples were provided for each theme/sub-theme. To account for reviewer comments unrelated to the IMRaD structure, a sub-category called ‘generic codes’ was created to collect more general comments. When reviewer comments could not be assigned to any of the existing themes/sub-themes, they were noted as “Miscellaneous”. Table 2 shows the final data extraction sheet including anchoring examples.

**Table 2:** Data extraction sheet used to extract free text comments from the reviewer’s report

Theme/sub-theme	Anchoring example	Example as taken from the reviewer’s report (copy & paste) Reviewer(s):
**DimensionI: Scientific/technical content**		
**GENERIC CODES**		
**Adding information/detail/nuances**	Indicate the number of people who refused to participate in the interviews.	
**Clarification needed**	Not clear if the word “prescriber” refers to GP or pharmacist or health professionals.	
**Justification required**	Justification for why this study is needed.	
**Further explanation required**	The mixed inductive/deductive analysis of data needs further explanation.	
**Erroneous/inaccurate information**	Introduction: UNAIDS isn’t a funding agency	
**Backing up claims**	Evidence that phenomenology was used is missing (except the author’s statement that they did so)	
**Unsubstantiated claims**		
**Discrepancy (e.g. between information presented in text and table)**	The text describing what the participants spent the money doesn’t jive with Table 2 (e.g. entertainment, dances, supporting parents is in the text but not reflected in the table).	
**Supporting reference(s) needed**		
**Inconsistency**	The research questions at the introduction are different from those at the Methods section	
**Provision of example(s)**	Provide examples to support theme “Evidence”	
**Confirmation/approval (from reviewer)**	The study uses mixed qualitative research methods which, in my opinion, are appropriate.	
**Inappropriate terminology**	Throughout the manuscript the term “elderly” is used, which is inappropriate as some older adults may find this term derogatory.	
**Internationalisation**	Clarify local context to make it understandable for an international readership.	
**Suggestion for literature**	Concrete suggestions for further literature were provided by the reviewer.	
**Description of table**	Table 1 gives a good description of your results, but I would suggest providing a short description of the table in the text with reference to this.	
**Observation/participant observation**	Put here any reviewer comments pertaining to direct or indirect (participant) observation as a data collection technique.	
**Miscellaneous**	Put here any reviewer comments that do not fit anywhere else!	
**INTRODUCTION**		
**Absence of important background information**	I would expect to see an overview of different models of care that use a team-based approach and references to the models that are mostly used in Europe.	
**Linking studies (‘Introduction’ section)**	Make clear link between larger “main” study and current paper (i.e. participants are from a sub-group of a larger study).	
**Putting information into context (‘Introduction’ section)**	The readers need to be aware of the Dutch context, the structure and organisation of the health and welfare system serving the elderly in the Netherlands.	
**Unclear research question**	This research question is unclear. Please rephrase.	
**METHODS**		
**Suggestion of what to call the method/methodology**	NOT phenomenology BUT descriptive qualitative analysis	
**Use of methodology-specific terminology**	Use of the term “lived experience” in phenomenology.	
**Lack of theoretical underpinning**	Complete absence of any theoretical framework that underpins the study.	
**Alignment to theory/framework**	Bandura’s self-efficacy is mentioned as a term but there is no alignment of the theory with the findings or the discussion section.	
**Training of researcher(s)**	Describe what training has been provided to researchers conducting the interviews	
**Recruitment of participants**	It is not clear exactly how the potential participants were identified and the method of recruitment, i.e., face-to-face, email.	
**Setting**	Setting - it is not clear if the participants are each from a distinct nursing home or not. Perhaps this could be included in Tables 1 and 2, e.g. were all four older people from the same home or each from a different home?	
**Sampling**	The authors need to set clear criteria for their purposeful sampling	
**Rational for sample size**		
**Small sample size**		
**Issues of participant anonymity**		
**Composition of sample**	There might be differences between medical and non-medical approaches to advance care planning.	
**Issues of bias**	Coding of transcripts by one researcher might introduce researcher bias	
**Interview guide (development, pre-test, etc.)**	You state that the interview guide was pre-tested on two RNs and one older person. Were data collected from these three individuals included in your final data set? Please include a statement about this in the manuscript.	
**Data saturation**	Saturation of themes/data	
**Details of analysis process**		
**Ethical considerations (e.g. ethical approval, etc.)**	You need to include something about ethical considerations in the Method section.	
**Reflexivity/Reflection**	Analyst relation to the data, especially relevant to a phenomenological approach (e.g. reflect on the impact of your own biases).	
**Quality criteria**	Issues of trustworthiness/member checking/respondent validation (e.g. participants to check if themes are correct).	
**RESULTS**		
**Counting in qualitative research**	use of relative terms such as “many”, “some”, “few” or “a handful”; attitude that qualitative research does not require counts	
**Data forcing**	Forcing your data to fit themes	
**Themes/sub-themes are not (sufficiently) supported by data**	It seems odd that this theme has no data supporting it from the comments made by older people.	
**Robust/rich data analysis**	Consider “deviant” or “appositional” viewpoints to get a richer analysis (e.g. reporting of “outliers”.	
**Results are quote heavy**	Be more selective about use of examples, i.e., do not provide an example to every theme/sub-theme.	
**Lengths of quotes (i.e. too short or too long)**		
**Quotes are inappropriate (e.g. too generic)**	The Results contains quite a bit of generic quotes such as “I think the majority of the time doctors don’t [provide enough education] and we’ll do it for them”, which could be applied to most conditions where pharmacists are being asked to play a more important role.	
**Opposed results**	Did you find something in the older people and nurses’ perspectives to be against each other?	
**Fit of data (in relation to the method)**		
**Development of a (new) model or framework (‘Results’ section)**	The analysis and conceptual development leads to a model of otherness, watchfulness and agency. It would be useful to know more about the process of selecting these three aspects as core. Was consideration given to additional/alternative aspects, and why were these rejected for example.	
**DISCUSSION & CONCLUSION**		
**Relate findings to (wider) literature**	Place discussion better within the context of existing research.	
**Putting information into context (‘Discussion’ section’)**	The readers need to be aware of the Dutch context, the structure and organisation of the health and welfare system serving the elderly in the Netherlands.	
**Highlighting differences in perspectives**	That would be fine to highlight differences in perspectives (i.e. older people and nurses’ perspectives) and discuss about them in the discussion.	
**Conflating of issues**		
**Development of a (new) model or framework (‘Discussion’ section)**	The analysis and conceptual development leads to a model of otherness, watchfulness and agency. It would be useful to know more about the process of selecting these three aspects as core. Was consideration given to additional/alternative aspects, and why were these rejected for example.	
**Transferability of findings**	“Generalisability” of the data if sample included only four participants	
**Implications for research/practice/ theory/teaching/etc.**	Provide implications for clinical nursing.	
**Recommendations for research/practice/theory/teaching/etc.**	Add suggestions for further studies/research.	
**Add strengths/limitations (of the study)**	Some of the quotes suggest to me the presence of social desirability bias, especially around the school attendance and the school - conditioned cash given that they all started out in school and especially since they turned out to be more likely to miss school!. No way to account for this but should be discussed in more detail in the limitations.	
**Backing up conclusions**		
**Unsubstantiated conclusions**		
**Conclusions do not reflect discussion section**	Conclusions: This section could be edited to further reflect the comments made above related to the discussion section.	
**REFERENCES**		
**Outdated references**	Use of outdated references, thus employ more recent evidence.	
**`Too few/too many references**	I would argue that the citation list is over-labored.	
**DimensionII: Writing criteria/Representation**		
**Language editing/proof reading**	The manuscript would benefit from English language editing.	
**Spelling/typos/omissions**	Words running into each other. Incomplete references.	
**Re-wording**	Consider re-wording the title.	
**Re-placing words**	For example in line 242 and 243 and I quote “Pharmacists viewed their main role to be providing advice and education to people with gout. Pharmacists demonstrated a good understanding of gout and how it is managed, which could facilitate their greater involvement in the management of people living with gout”. The second pharmacist could be replaced by “They”, considering that the first sentence introduces it.	
**Readability**	Moving content from one place to another to enhance readability (e.g. removing the reference to Table 1 from the Methods and placing it at the beginning of the Results section).	
**Concise writing**	Clear statement of findings/implications.	
**Structure**	I suggest moving the first sentence about the sociological perception of cancer after an objective quantification of the cancer burden and of PCA burden.	
**Follow journal’s reference style and/or instructions for authors**	The paper needs to follow the reference style of the journal as well as the instruction of the authors.	
**Personalised terms (i.e. subjective/objective style of writing)**	There is too much use of personalized terms in the text such as ‘we’ and/or ‘our’.	
**Mode of representation**	Rather than putting it in writing, the information would be better represented as a flow chart.	
**Lengths of manuscript (i.e. either too long or too short)**	Some of the discussion/conclusions becomes a bit verbose/repetitive. Is it possible to cut that down at all without losing the empirical grounding and relevant context?	
**DimensionIII: TechnicalCriteria**		
**(Re-)submission of manuscript**	Please include all comments for the authors in this box rather than uploading your report as an attachment. Please only upload as attachments annotated versions of manuscripts, graphs, supporting materials or other aspects of your report which cannot be included in a text format. Please overwrite this text when adding your comments to the authors.	

### Data extraction procedure

Data extraction was accomplished by six doctoral students (coders). On average, each coder was allocated 18 articles. After reading the reviews, coders independently classified each comment using the classification system. In line with Day et al. [30] a reviewer comment was defined as “*a distinct statement or idea found in a review, regardless of whether that statement was presented in isolation or was included in a paragraph that contained several statements.*” Editor comments were not included. Reviewers’ comments were copied and pasted into the most appropriate item of the classification system following a set of pre-defined guidelines. For example, a reviewer comment could only be coded once by assigning it to the most appropriate theme/sub-theme. A separate data extraction sheet was used for each article. For the purpose of calibration, the first completed data extraction sheet from each coder together with the reviewer’s comments was sent to the study coordinator (ORH) who provided feedback on classifying the reviewer comments. The aim of the calibration was to ensure that all coders were working within the same parameters of understanding, to discuss the subtleties of the judgement process and create consensus regarding classifications. Although the assignment to specific themes/sub-themes is, by nature, a subjective process, difficult to assign comments were classified following discussion and agreement between coder and study coordinator to ensure reliability. Once all data extraction was completed, two experienced qualitative researchers (CB-J, JT) independently undertook a further calibration exercise of a random sub-sample of 20% of articles (*n* = 22) to ensure consistency across coders. Articles were selected using a random number generator. For these 22 articles, classification discrepancies were resolved by consensus between coders and experienced researchers. Finally, all individual data extraction sheets were collated to create a comprehensive Excel spreadsheet with over 8000 cells that allowed tallying the reviewer’s comments across manuscripts for the purpose of data analysis. For each manuscript, a reviewer could have several remarks related to one type of comment. However, each type of comment was scored only once per category.

Finally, reviewer comments were ‘quantitized’ [31] by applying programming language (Python) to Jupyter Notebook, an open-source web application, to perform frequency counts of free-text comments regarding the 77 items. Among other data manipulation, we sorted elements of arrays in descending order of frequency using Pandas, counted the number of studies in which a certain theme/sub-theme occurred, conducted distinct word searches using NLTK 3 or grouped data according to certain criteria. The calculation of frequencies is a way to unite the empirical precision of quantitative research with the descriptive precision of qualitative research [32]. This quantitative transformation of qualitative data allowed extracting more meaning from our spreadsheet through revealing patterns across themes/sub-themes, thus giving indicators about which of them to analyse using thematic analysis.

## Results

A total of 109 manuscripts submitted to nine open-access journals were included in the FMRS. When scrutinising the peer review reports, we noticed that on one occasion the reviewer’s comments were missing [33]. For the remaining 108 manuscripts, reviewer comments were accessible via the journal’s pre-publication history. On close inspection, however, it became apparent that one article did not contain qualitative research, thus leaving ultimately 107 articles to work with (supplementary file). Considering that each manuscript could potentially be reviewed by multiple reviewers and underwent at least one round of revision, the total number of reviewer reports analysed amounted to 347 containing collectively 1703 reviewer comments. The level of inter-rater agreement for the 22 articles included in the calibration exercise was 97%. Disagreement was, for example, in relation to coding a comment as “miscellaneous” or as “confirmation/approval (from reviewer)”. For 18 out of 22 articles, there was 100% agreement for all types of comments.

### Variation in number of reviewers

The number of reviewers invited by the editor to review a submitted manuscript varied greatly within and among journals. While the majority of manuscripts across journals had been reviewed by two to three reviewers, there were also significant variations. For example, the manuscript submitted to *BMC Medical Education* by Burgess et al. [34] had been reviewed by five reviewers whereas the manuscript submitted to *BMC Public Health* by Lee and Lee [35] had been reviewed by one reviewer only. Even within journals there was a huge variation. Among our sample, *BMC Public Health* had the greatest variance ranging from one to four reviewers. Besides, it was noted that additional reviewers were called in not until the second or even third revision of the manuscript. A summary of key information on journals included in the FMRS is provided in Table 3.

**Table 3:** Summary of key information on open access journals included in the FMRS

Journal	Number of qualitative articles published during 1 Jan – 31 March 2018	Number of revisions (mode; min. – max.)	Number of reviewers (mode; min. – max.)
BMC Complementary and Alternative Medicine	3	1; 1–2	2; 2–2
BMC Family Practice	11	1; 1–2	2; 2–4
BMC Health Services Research	41	2; 1–4	2; 1–4
BMC Medical Education	7	1; 1–2	2; 2–5
BMC Medical Ethics	5	1; 1–4	2; 2–3
BMC Nursing	2* (*there were actually three papers published in this timeframe; however, reviewer comments for one paper was not available online)	n/a; 1–2	n/a; 2–3
BMC Public Health	29	2; 1–2	2; 1–4
Health Research Policy and Systems	6	1; 1–1	2; 2–3
Implementation Science	3	1; 1–3	2; 2–3
**Total**	**107**		

### “Quantitizing” reviewer comments

The frequency analysis revealed that the number of articles in which a certain theme/sub-theme occurred ranged from 1 to 79. Across all 107 articles, the types of comments most frequently reported were in relation to generic themes. Reviewer comments regarding “Adding information/detail/nuances”, “Clarification needed”, “Further explanation required” and “Confirmation/approval (from reviewer)” were used in 79, 79, 66 and 63 articles, respectively. The four most frequently used themes/sub-themes are composed of generic codes from dimension I (“Scientific/technical content”). Leaving all generic codes aside, it became apparent that among the 30 most frequently employed themes “Writing criteria” (dimension II) is the top ranking theme, followed by comments in relation to the “Methods” (dimension I) (Table 4).

**Table 4:** The 30 most frequently used themes reviewers provided feedback to (in descending order)

Theme/sub-theme	Dimension/Code	Number of articles in which theme occurred
Adding information/detail/nuances	Dimension I/generic code	79
Clarification needed	Dimension I/generic code	79
Further explanation required	Dimension I/generic code	66
Confirmation/approval (from reviewer)	Dimension I/generic code	63
Details of analysis process	Dimension I/methods	60
Miscellaneous	Dimension I/generic code	58
Structure	Dimension II/writing criteria	54
Re-wording	Dimension II/writing criteria	53
Add strengths/imitations (of the study)	Dimension I/discussion & conclusion	49
Absence of important background information	Dimension I/introduction	48
Language editing/proof reading	Dimension II/writing criteria	42
Relate findings to (wider) literature	Dimension I/discussion & conclusion	42
Composition of sample	Dimension I/methods	41
Spelling/typos/omissions	Dimension II/writing criteria	40
Sampling	Dimension I/methods	34
Justification required	Dimension I/generic code	33
Interview guide (development, pre-test)	Dimension I/methods	32
Re-placing words	Dimension II/writing criteria	32
Putting information into context	Dimension I/discussion & conclusion	32
Robust/rich data analysis	Dimension I/results	30
Concise writing	Dimension II/writing criteria	29
Putting information into context	Dimension I/introduction	29
Recruitment of participants	Dimension I/methods	28
Setting	Dimension I/methods	27
Inconsistency	Dimension I/generic code	27
Mode of representation	Dimension II/writing criteria	26
Implications for research/practice/theory/teaching etc.	Dimension I/discussion & conclusion	25
Suggestion for literature	Dimension I/generic code	24
Readability	Dimension II/writing criteria	24
Supporting reference(s) needed	Dimension I/generic code	24

Subsequently, we present key qualitative findings regarding “Confirmation/approval from reviewers” (generic), “Sampling” and “Analysis process” (methods), “Robust/rich data analysis and “Themes/sub-themes” (results) as well as findings that suggest an underlying quantitative mindset of the reviewers.

### Confirmation/approval from reviewers (generic)

The theme “confirmation/approval from reviewers” ranks third among the top 30 categories. A total of 63 manuscripts contained at least one reviewer comment related to this theme. Overall, reviewers maintained a respectful and affirmative rhetoric when providing feedback. The vast majority of reviewers began their report by stating that the manuscript was well written. The following is a typical example:

*“Overall, the paper is well written, and theoretically informed.”***Article #14.**

Reviewers then continued to add explicit praise for aspects or sections that were particularly innovative and/or well constructed before they started to put forward any negative feedback.

### Sampling (methods)

Across all 107 articles there were 34 reviewer comments in relation to the sampling technique(s). Two major categories were identified: (1) composition of the sample and (2) identification and justification of selected participants. Regarding the former, reviewers raised several concerns about how the sample was composed. For instance, one reviewer wanted to know the reason for female predominance in the study and why an entire focus group was composed of females only. Another reviewer expressed strong criticism on the composition of the sample since only young, educated and non-minority white British participants were included in the study. The reviewer commented:

“*So a typical patient was young, educated and non-minority White British? The research studies these days should be inclusive of diverse types of patients and excluding patients because of their age and ethnicity is extremely concerning to me. This assumption that these individuals will “find it more difficult to complete questionnaires” is concerning*” **Article #40.**

This raised concerns of potentially excluding important diverse perspectives – such as extreme or deviant cases – from other participants. Similarly, some reviewers expressed concerns that relevant groups of people were not interviewed, calling into question that the findings were theoretically saturated. In terms of the identification of participants, reviewers raised questions regarding how the authors obtained the necessary characteristics to achieve purposive sampling or why only certain groups of people were included for interviews. Besides that, reviewers criticised that some authors did not mention their inclusion/exclusion criteria for selecting participants or did not specify their sampling method. For example:

*“The authors state that they recruited a purposive sample of patients for the interviews. Concerning which variables was this sampling purposive? Are there any studies informing the patient selection process?”***Article #61.**

Hence, reviewers requested more detailed information on how participants were selected and to clearly state the type of sampling. Apart from the two key categories, reviewers made additional comments in relation to data saturation, transferability of findings, limitations of certain sampling methods and criticised the lack of description of participants who were approached but refused to participate in the study.

### Details of analysis process (methods)

In 60 out of 107 articles, reviewers made comments in relation to the data analysis. The vast majority of comments stressed that authors provided scarce information about the analysis process. Hence, reviewers requested a more detailed description of the specific analysis techniques employed so that readers can obtain a better understanding of how the analysis was done to judge the trustworthiness of the findings. To this end, reviewers frequently requested an explicit statement on whether the analysis was inductive or deductive or iterative or sequential. One reviewer wrote the following comment:

*“Please elaborate more on the qualitative analysis. The authors indicate that they used ‘iterative’ approaches. While this is certainly laudable, it is important to know how they moved from codes to themes (e.g. inductively? deductively?)”***Article #5.**

Since there are many approaches to analysing qualitative data, reviewers demanded sufficient detail in relation to the underlying theoretical framework used to develop the coding scheme, the analytic process, the researchers’ background (e.g. profession), the number of coders, data handling, length of interviews and whether data saturation occurred. Over a dozen reviewer comments were specifically in relation to the identification of themes/sub-themes. Reviewers requested a more detailed description on how the themes/sub-themes were derived from codes and whether they were developed by a second researcher working independently from each other.

*“I would have liked to read how their themes were generated, what they were and how they assured robust practices in qualitative data analysis”.***Article #43.**

Besides that, some reviewers were in the opinion that the approach to analysis has led to a surface-level penetration of the data which was reflected in the Results section where themes were underexplored (for more detail see “*Robust/rich data analysis”* below). Finally, reviewer comments that occurred infrequently included questions concerning the inter-rater reliability, competing interpretations of data, the use of computer software or the original interview language.

### Robust/rich data analysis (results)

Among the 30 reviewer comments related to this theme/sub-theme, three key facets were observed: (1) greater analytical depth required, (2) suggestions for further analysis, and (3) themes are underexplored. In relation to the first point, reviewers requested more in-depth data analysis to strengthen the quality of the manuscript. Reviewers were in the opinion that authors reproduced interview data (raw data) in a reduced form with minimal or no interpretation, thus leaving the interpretation to the reader. Other reviewers referred to manuscripts as preliminary drafts that need to be further analysed to achieve greater analytical depth of themes, make links between themes or identify variations between respondents. In relation to the second point, several reviewers offered suggestions for further analysis. They provided detailed information on how to further explore the data and what additional results they would like to see in the revised version (e.g. group comparison, gender analysis). The latter aspect goes hand in hand with the third point. Several reviewers pointed out that the findings were shallow, simplistic or superficial at best; lacking the detailed descriptions of complex accounts from participants. For example:

*“The results of the study are mostly descriptive and there is limited analysis. There is also absence of thick description, which one would expect in a qualitative study”.***Article #34.**

Even after the first revision, some manuscripts still lacked detailed analysis as the following comment from the same reviewer illustrates:

*“I believe that the results in the revised version are still mostly descriptive and that there is limited analysis”.***Article #34, R1.**

Other, less frequently mentioned reviewer comments included lack of deviant cases or absence of relationships between themes.

### Themes/sub-themes (results)

In total, there were 24 reviewer comments in relation to themes/sub-themes. More than half of the comments fell into one of the three categories: (1) themes/sub-themes are not sufficiently supported by data, (2) example/excerpt does not fit the stated theme, and (3) use of insufficient quotes to support theme/sub-theme. In relation to the first category, reviewers largely criticised that the data provided were insufficient to warrant being called a theme. Reviewers requested to provide data *“from more than just one participant”* to substantiate a certain theme or criticised that only a short excerpt was provided to support a theme. The second category dealt with reviewer comments that questioned whether the excerpts provided actually reflected the essence of a theme/sub-theme presented in the results section. The following reviewer comment exemplifies the issue:

*“The data themes seem valid, but the data and narratives used to illustrate that don’t seem to fit entirely under each sub-heading”.***Article #99.**

Some reviewers provided alternative suggestions on how to call a theme/sub-theme or advised the authors to rethink if excerpts might be better placed under a different theme. The third category concerns themes/sub-themes that are not sufficiently supported by participants’ quotes. Reviewers perceived direct quotes as evidence to support a certain theme or as a means to add strength to the theme as the following example illustrates:

*“Please provide at least one quote from each school leader and one quote from children to support this theme, if possible. It would seem that most, if not all, themes should reflect data from each participant group”.***Article #88.**

Hence, the absence of quotes prompted reviewers to request at least one quote to justify the existence of that theme. The inclusion of a rich set of quotes was perceived as strength of a manuscript. Finally, less frequently raised reviewer comments related to the discrimination of similar themes, the presentation of quotes in tables (rather than under the appropriate theme headings), the lack of defining a theme and reducing the number of themes.

### Quantitative mindset

Some reviewers who were appointed by journal editors to review a manuscript containing qualitative research evaluated the quality of the manuscript from a perspective of a quantitative research paradigm. Some reviewers not only used terminology that is attuned to quantitative research, but also their judgements were based on a quantitative mindset. In particular, there were a number of reviewer comments published in *BMC Health Services Research*, *BMC Medical Education* and *BMC Family Practice* that demonstrated an apparent lack of understanding of the principles underlying qualitative inquiry of the person providing the review. First, several reviewers seemed to have confused the concept of generalisability with the concept of representativeness inherently associated with the positivist tradition. For instance, reviewers erroneously raised concerns about whether interviewees were “representative” of the “final target population” and requested the provision of detailed demographic characteristics.

*“Need to better describe how the patients are representative of patients with chronic heart failure in the Netherlands generally. The declaration that “a representative group of patients were recruited” would benefit from stating what they were representative of.”***Article # 66.**

Similarly, another reviewer wanted to know from the authors how they ensured that the qualitative analysis was done objectively.

*“The reader would benefit from a detailed description of […] how did the investigators ensure that they were objective in their analysis – objectivity and trustworthiness?”***Article #22.**

Furthermore, despite the fact that the paradigm wars have largely come to an end, hostility has not ceased on all fronts. In some reviewers the dominance and superiority of the quantitative paradigm over the qualitative paradigm is still present as the following comment illustrates:

*“The main question and methods of this article is largely qualitative and does not seem to have significant implications for clinical practice, thus it may not be suitable to publish in this journal.”***Article #45.**

Finally, one reviewer apologised at the outset of the reviewer’s report for being unable to judge the data analysis due to the absence of sufficient knowledge in qualitative research.

## Discussion

Overall, in this FMRS we found that reviewers maintained a respectful and affirmative rhetoric when providing feedback. Yet, the positive feedback did not overshadow any key negative points that needed to be addressed in order to increase the quality of the manuscript. However, it should not be taken for granted that all reviewers are as courteous and generous as the ones included in our particular review, because as Taylor and Bradbury-Jones [36] observed there are many examples where reviewers can be unhelpful and destructive in their comments.

A key finding of this FMRS is that reviewers are more inclined to comment on the writing rather than the methodological rigour of a manuscript. This is a matter of concern, because Altman [37] – the originator of the EQUATOR (Enhancing the Quality and Transparency of Health Research) Network – has pointed out: “Unless methodology is described the conclusions must be suspect”. If we are to advance the quality of qualitative research then we need to encourage clarity and depth in reporting the rigour of research.

When reviewers did comment on the methodological aspects of an article, issues frequently commented on by reviewers were in relation to sampling, data analysis, robust/rich data analysis as reflected in the findings and themes/sub-themes that are insufficiently supported. Considerable work has been undertaken over the past decade trying to improve the reporting standards of qualitative research through the dissemination of qualitatively oriented reporting guidelines such as the ‘Standards for Reporting Qualitative Research’ (SRQR) [38] or the ‘Consolidated Criteria for Reporting Qualitative Research’ (COREQ) [39] with the aim of improving transparency of qualitative research. Although these guidelines appear to be comprehensive, some important issues identified in our study are not mentioned or only dealt with somewhat superficially: sampling for example. Neither COREQ nor SRQR shed light on the appropriateness of the sample composition, i.e., to critically question whether all relevant groups of people have been identified as potential participants or whether extreme or deviant cases were sought.

Similarly, lack of in-depth data analysis has been identified as another weakness where uninterpreted (raw) data were presented as if they were findings. However, existing reporting guidelines are not sharp enough to distinguish between findings and data. While findings are researchers’ interpretations of the data they collected, data consist of empirical, uninterpreted material researchers offer as their findings [32]. Hence, we suggest modifying the current reporting guidelines by including a further item to the checklist called “Degree of data transformation”. The suggested checklist item might prompt both authors and reviewers to make a judgment about the degree to which data have been transformed, i.e., interpretively removed from data as given. The rationale for the new item is to raise authors’ and reviewers’ awareness for the appropriateness of the degree of data transformation in relation to the chosen analysis method. For example, findings derived from content analysis remain close to the data as they were given to the research; they are often organised into surface classification systems and summarised in brief text. Findings derived from grounded theory, however, should offer a coherent model or line of argument which addresses causality or the fundamental nature of events or experiences [32].

Besides that, some reviewers put forward comments that we refer to as aligning with a ‘quantitative mindset’. Such reviewers did not appear to understand that rather than aspiring to statistical representativeness, in qualitative research participants are selected purposefully for the contribution they can make towards the phenomenon under study [40]. Hence, the generalisability of qualitative findings beyond an immediate group of participants is judged by similarities between the time, place, people or other social contexts [41] rather than in relation to the comparability of the demographic variables. It is the fit of the topic or the comparability of the problem that is of concern [40].

The majority of issues that reviewers picked up on are already mentioned in reporting guidelines, so there is no reason why these were omitted by researchers. Many journals now insist on alignment with COREQ criteria, so there is an important question to be asked as to why this is not always happening. We suggest that completion of an established reporting checklist (e.g. COREQ, SRQR) on submission becomes a requirement.

In this FMRS we have made judgements about fellow peer reviewers and found their feedback to be constructive, but also, among some, we found some lack of grasp of the essence of the qualitative endeavor. Some reviewers did not seem to understand that objectivity and representative sampling are the antithesis of subjectivity, reflexivity and data saturation. We acknowledge though, that individual reviewers might have varying levels of experience and competence both in terms of qualitative research, but also in the reviewing process. We found one reviewer who apologised at the outset of the reviewer’s report for being unable to judge the data analysis due to their absence of sufficient knowledge in qualitative research. In line with Spigt and Arts [42], we appreciate the honesty of that reviewer for being transparent about their skillset. The lessons here we feel are for more experienced reviewers to offer support and reviewing mentorship to those who are less experienced and for reviewers to emulate the honesty of the reviewer as discussed here, by being open about their capabilities within the review process.

Based on our findings, we have a number of recommendations for both researchers and reviewers. For researchers reporting qualitative studies, we suggest that particular attention is paid to reporting of sampling techniques, both in the characteristics and composition of the sample, and how participants were selected. This is an issue that the reviewers in our FMRS picked up on, so forewarned is forearmed. But it is also crucially important that sampling matters are not glossed over, so this constitutes good practice in research reporting as well. Second, it seems that qualitative researchers do not give sufficient detail about analytic techniques and underlying theoretical frameworks. The latter has been pointed out before [25], but both these aspects were often the subject of reviewer comments.

Our recommendation for reviewers is simply to be honest. If qualitative research is not an area of expertise, then it is better to decline to undertake the review, than to apply a quantitative lens in the assessment of a qualitative piece of work. It is inappropriate to ask for details about validity and generalisability and shows a lack of respect to qualitative researchers. We are well beyond the arguments about quantitative versus qualitative [43]. It is totally appropriate to comment on background and findings and any obvious deficiencies. Finally, our recommendation to editors is a difficult one, because as editors ourselves we know how challenging it can be to find willing reviewers. When selecting reviewers however, it is as important to bear in mind the methodological aspects of an article and its subject, and to select reviewers with appropriate methodological expertise. Some journals make it a requirement for quantitative articles to be reviewed by a statistical expert and we think this is good practice. When it comes to qualitative articles however, the methodological expertise of reviewers may not be so stringently noted and applied. Editors could make a difference here and help to push up the quality of qualitative reviews.

### Strengths and weaknesses

Since we had only access to reviewer’s comments of articles that were finally published in open access journals, we are unable to compare them to types of comments related to rejected submissions. Thus, this study was limited to manuscripts that were sent out for external peer review and were finally published. Furthermore, the chosen study design of analysing only reviewer comments of published articles with an open system of peer review did not allow direct comparison with reviewer comments derived from blind-review.

FMRS provides a snap-shot of a particular issue at one particular time [23]. To that end, findings might be different in another review undertaken in a different time period. However, as a contemporary profile of reviewing within qualitative research, the current findings provide useful insights for authors of qualitative reports and reviewers alike. Further research should focus on comparing reviewer comments taken from an open and closed system of peer review in order to identify similarities and differences between the two models of peer review.

A limitation is that we reviewed open access journals because this was the only way of accessing a range of comments. The alternative that we did consider was to use the feedback provided by reviewers on our own manuscripts. However, this would have lacked the transparency and traceability associated with this current FMRS, which we consider to be a strength. That said, there may be an inherent problem in having reviewed open access peer review comments, where both the author and reviewer are known. Reviewers are unable to ‘hide behind’ the anonymity of blind peer review and this might reflect, at least in part, why their comments as analysed for this review were overwhelmingly courteous and constructive. This is at odds with the comments that one of us has received as part of a blind peer review: ‘silly, silly, silly’ [36].

## Conclusions

This FMRS has highlighted some important issues in the field of qualitative reviewing that hold lessons for authors, reviewers and editors. Authors of qualitative reports are called upon to follow guidelines on reporting and any amendments that these might contain as recommended by the findings of our review. Humility and transparency are required among reviewers when it comes to accepting to undertake a review and an honest appraisal of their capabilities in understanding the qualitative endeavor. Journal editors can assist this by thoughtful and judicious selection of reviewers. Ultimately, all those involved with the publication process can drive up the quality of individual qualitative articles and the synergy is such that this can make a significant impact on quality across the field.

## Supplementary information

**Additional file 1 **References of all manuscripts included in the analysis (*n* = 107).

## Data Availability

The datasets used and/or analysed during the current study are available from the corresponding author on reasonable request.

## Abbreviations

BMC: BioMed central
BMJ: British medical journal
COREQ: Consolidated criteria for reporting qualitative research
EQUATOR: Enhancing the quality and transparency of health research
FMRS: Focused mapping review and synthesis
IMRaD: Introduction, methods, results and discussion
NLTK: Natural language toolKit
SRQR: Standards for reporting qualitative research
