# Supplementary material for: What feedback do reviewers give when reviewing qualitative manuscripts? A focused mapping review and synthesis
Source: BMC Med Res Methodol. 2020 May 18;20:122. doi: 10.1186/s12874-020-01005-y (PMC7236308; doi:10.1186/s12874-020-01005-y)
Supplement: Supplementary file 1 — Additional file 1 References of all manuscripts included in the analysis (n = 107). [file 12874_2020_1005_MOESM1_ESM.docx]

**Supplementary file:** References of all manuscripts included in the analysis (n=107)

1. Welz AN, Emberger-Klein A, Menrad K2. Why people use herbal medicine: insights from a focus-group study in Germany. BMC Complement Altern Med. 2018;18:92.
2. Haque MI, Chowdhury ABMA, Shahjahan M, Harun MGD. Traditional healing practices in rural Bangladesh: a qualitative investigation. BMC Complement Altern Med. 2018;18:62.
3. Zörgő S, Purebl G, Zana Á. A qualitative study of culturally embedded factors in complementary and alternative medicine use. BMC Complement Altern Med. 2018;18:25.
4. Mouton Dorey C, Baumann H, Biller-Andorno N. Patient data and patient rights: Swiss healthcare stakeholders' ethical awareness regarding large patient data sets - a qualitative study. BMC Med Ethics. 2018;19:20.
5. Ignatowicz A, Slowther AM, Elder P, Bryce C, Hamilton K, Huxley C, Forjaz V, Sturt J, Griffiths F. Ethical implications of digital communication for the patient-clinician relationship: analysis of interviews with clinicians and young adults with long term conditions (the LYNC study). BMC Med Ethics. 2018;19:11.
6. Tuffrey-Wijne I, Curfs L, Finlay I, Hollins S. Euthanasia and assisted suicide for people with an intellectual disability and/or autism spectrum disorder: an examination of nine relevant euthanasia cases in the Netherlands (2012-2016). BMC Med Ethics. 2018;19:17.
7. Staunton C, Tindana P, Hendricks M, Moodley K. Rules of engagement: perspectives on stakeholder engagement for genomic biobanking research in South Africa. BMC Med Ethics. 2018;19:13.
8. Span-Sluyter CAMFH, Lavrijsen JCM, van Leeuwen E, Koopmans RTCM. Moral dilemmas and conflicts concerning patients in a vegetative state/unresponsive wakefulness syndrome: shared or non-shared decision making? A qualitative study of the professional perspective in two moral case deliberations. BMC Med Ethics. 2018;19:10.
9. Deeming S, Reeves P, Ramanathan S, Attia J, Nilsson M, Searles A. Measuring research impact in medical research institutes: a qualitative study of the attitudes and opinions of Australian medical research institutes towards research impact assessment frameworks. Health Res Policy Syst. 2018;16:28.
10. Moore G, Redman S, Rudge S, Haynes A. Do policy-makers find commissioned rapid reviews useful? Health Res Policy Syst. 2018;16:17.
11. Wu S, Legido-Quigley H, Spencer J, Coker RJ, Khan MS. Designing evaluation studies to optimally inform policy: what factors do policy-makers in China consider when making resource allocation decisions on healthcare worker training programmes? Health Res Policy Syst. 2018;16:16.
12. Sriram V, Bennett S, Raman VR, Sheikh K. Developing the National Knowledge Platform in India: a policy and institutional analysis. Health Res Policy Syst. 2018;16:13.
13. Ezeanolue EE, Menson WNA, Patel D, Aarons G, Olutola A, Obiefune M, Dakum P, Okonkwo P, Gobir B, Akinmurele T, Nwandu A, Khamofu H, Oyeledun B, Aina M, Eyo A, Oleribe O, Ibanga I, Oko J, Anyaike C, Idoko J, Aliyu MH, Sturke R; Nigeria Implementation Science Alliance. Gaps and strategies in developing health research capacity: experience from the Nigeria Implementation Science Alliance. Health Res Policy Syst. 2018;16:10.
14. Lehn SF, Thuesen J, Bunkenborg G, Zwisler AD, Rod MH. Implementation between text and work-a qualitative study of a readmission prevention program targeting elderly patients. Implement Sci. 2018;13:38.
15. Raven J, Baral S, Wurie H, Witter S, Samai M, Paudel P, Subedi HN, Martineau T, Elsey H, Theobald S. What adaptation to research is needed following crises: a comparative, qualitative study of the health workforce in Sierra Leone and Nepal. Health Res Policy Syst. 2018;16:6.
16. Stephan AJ, Kovacs E, Phillips A, Schelling J, Ulrich SM, Grill E. Barriers and facilitators for the management of vertigo: a qualitative study with primary care providers. Implement Sci. 2018;13:25.
17. Nilsen P, Wallerstedt B, Behm L, Ahlström G. Towards evidence-based palliative care in nursing homes in Sweden: a qualitative study informed by the organizational readiness to change theory. Implement Sci. 2018;13:1.
18. Berglas NF, Roberts SCM. The development of facility standards for common outpatient procedures and implications for the context of abortion. BMC Health Serv Res. 2018;18:212.
19. Brindle ME, Henrich N, Foster A, Marks S, Rose M, Welsh R, Berry W. Implementation of surgical debriefing programs in large health systems: an exploratory qualitative analysis. BMC Health Serv Res. 2018;18:210.
20. Karstens S, Kuithan P, Joos S, Hill JC, Wensing M, Steinhäuser J, Krug K, Szecsenyi J. Physiotherapists' views of implementing a stratified treatment approach for patients with low back pain in Germany: a qualitative study. BMC Health Serv Res. 2018;18:214.
21. O'Sullivan GA, Hanlon C, Dentry T, Morris T, Banting L. A qualitative exploration of the client experience of inter-professional practice in the delivery of ActivePlus: a combined smoking cessation and physical activity intervention. BMC Health Serv Res. 2018;18:195.
22. Nwameme AU, Tabong PT, Adongo PB. Implementing Community-based Health Planning and Services in impoverished urban communities: health workers' perspective. BMC Health Serv Res. 2018;18:186.
23. Gustavsson C, Nordqvist M, Bröms K, Jerdén L, Kallings LV, Wallin L. What is required to facilitate implementation of Swedish physical activity on prescription? - interview study with primary healthcare staff and management. BMC Health Serv Res. 2018;18:196.
24. Theodoulou I, Reddy AM, Wong J. Is innovative workforce planning software the solution to NHS staffing and cost crisis? An exploration of the locum industry. BMC Health Serv Res. 2018;18:188.
25. Zullig LL, Goldstein KM, Bosworth HB, Andrews SM, Danus S, Jackson GL, Provenzale D, Weinberger M, Kelley MJ, Voils CI. Chronic disease management perspectives of colorectal cancer survivors using the Veterans Affairs healthcare system: a qualitative analysis. BMC Health Serv Res. 2018;18(1):171.
26. Johansen ML, Ervik B. Teamwork in primary palliative care: general practitioners' and specialised oncology nurses' complementary competencies. BMC Health Serv Res. 2018;18:159.
27. Braaf S, Ameratunga S, Nunn A, Christie N, Teague W, Judson R, Gabbe BJ. Patient-identified information and communication needs in the context of major trauma. BMC Health Serv Res. 2018;18:163.
28. Muzyamba C, Groot W, Tomini S, Pavlova M. Community mobilization and maternal Care of Women Living with HIV in poor settings: the case of Mfuwe, Zambia. BMC Health Serv Res. 2018;18:155.
29. Onarheim KH, Sisay MM, Gizaw M, Moland KM, Norheim OF, Miljeteig I. Selling my sheep to pay for medicines - household priorities and coping strategies in a setting without universal health coverage. BMC Health Serv Res. 2018;18:153.
30. Pennathur PR, Ayres BS. A qualitative investigation of healthcare workers' strategies in response to readmissions. BMC Health Serv Res. 2018;18:138.
31. Sabater-Hernández D, Tudball J, Ferguson C, Franco-Trigo L, Hossain LN, Benrimoj SI. A stakeholder co-design approach for developing a community pharmacy service to enhance screening and management of atrial fibrillation. BMC Health Serv Res. 2018;18:145.
32. Humphries N, Crowe S, Brugha R. Failing to retain a new generation of doctors: qualitative insights from a high-income country. BMC Health Serv Res. 2018;18:144.
33. Loss J, Weigl J, Ernstberger A, Nerlich M, Koller M, Curbach J. Social capital in a regional inter-hospital network among trauma centers (trauma network): results of a qualitative study in Germany. BMC Health Serv Res. 2018;18:137.
34. Carlfjord S, Öhrn A, Gunnarsson A. Experiences from ten years of incident reporting in health care: a qualitative study among department managers and coordinators. BMC Health Serv Res. 2018;18:113.
35. Mayes ME, Wilkinson C, Kuah S, Matthews G, Turnbull D. Change in practice: a qualitative exploration of midwives' and doctors' views about the introduction of STan monitoring in an Australian hospital. BMC Health Serv Res. 2018;18:119.
36. Khoza N, Stadler J, MacPhail C, Chikandiwa A, Brahmbhatt H, Delany-Moretlwe S. Cash transfer interventions for sexual health: meanings and experiences of adolescent males and females in inner-city Johannesburg. BMC Public Health. 2018;18:120.
37. Babac A, Frank M, Pauer F, Litzkendorf S, Rosenfeldt D, Lührs V, Biehl L, Hartz T, Storf H, Schauer F, Wagner TOF, Graf von der Schulenburg JM. Telephone health services in the field of rare diseases: a qualitative interview study examining the needs of patients, relatives, and health care professionals in Germany. BMC Health Serv Res. 2018;18:99.
38. Kpobi L, Swartz L, Ofori-Atta AL. Challenges in the use of the mental health information system in a resource-limited setting: lessons from Ghana. BMC Health Serv Res. 2018;18:98.
39. Wang X, Jiang R, Li J, Chen J, Burström B, Burström K. What do patients care most about in China's public hospitals? Interviews with patients in Jiangsu Province. BMC Health Serv Res. 2018;18:97.
40. Murphy M, Hollinghurst S, Salisbury C. Qualitative assessment of the primary care outcomes questionnaire: a cognitive interview study. BMC Health Serv Res. 2018;18:79.
41. Cataldo F, Seeley J, Nkhata MJ, Mupambireyi Z, Tumwesige E, Gibb DM; Lablite team. She knows that she will not come back: tracing patients and new thresholds of collective surveillance in PMTCT Option B. BMC Health Serv Res. 2018;18:76.
42. Pedersen MS, Landheim A, Møller M, Lien L. Acting on audit & feedback: a qualitative instrumental case study in mental health services in Norway. BMC Health Serv Res. 2018;18:71.
43. Chatwin J, Ackers L. Organisational barriers to the facilitation of overseas volunteering and training placements in the NHS. BMC Health Serv Res. 2018;18:69.
44. Altman L, Zurynski Y, Breen C, Hoffmann T, Woolfenden S. A qualitative study of health care providers' perceptions and experiences of working together to care for children with medical complexity (CMC). BMC Health Serv Res. 2018;18:70.
45. Nilsson G, Hansson K, Tiberg I, Hallström I. How dislocation and professional anxiety influence readiness for change during the implementation of hospital-based home care for children newly diagnosed with diabetes - an ethnographic analysis of the logic of workplace change. BMC Health Serv Res. 2018;18:61.
46. Chabot C, Gilbert M, Haag D, Ogilvie G, Hawe P, Bungay V, Shoveller JA. Anticipating the potential for positive uptake and adaptation in the implementation of a publicly funded online STBBI testing service: a qualitative analysis. BMC Health Serv Res. 2018;18:57.
47. Holt DH, Rod MH, Waldorff SB, Tjørnhøj-Thomsen T. Elusive implementation: an ethnographic study of intersectoral policymaking for health. BMC Health Serv Res. 2018;18:54.
48. Kiriazova T, Postnov O, Bingham T, Myers J, Flanigan T, Vitek C, Neduzhko O. Patient and provider perspectives inform an intervention to improve linkage to care for HIV patients in Ukraine. BMC Health Serv Res. 2018;18:58.
49. Mateo KF, Berner NB, Ricci NL, Seekaew P, Sikerwar S, Tenner C, Dognin J, Sherman SE, Kalet A, Jay M. Development of a 5As-based technology-assisted weight management intervention for veterans in primary care. BMC Health Serv Res. 2018;18:47.
50. Gupta A, Fledderjohann J, Reddy H, Raman VR, Stuckler D, Vellakkal S. Barriers and prospects of India's conditional cash transfer program to promote institutional delivery care: a qualitative analysis of the supply-side perspectives. BMC Health Serv Res. 2018;18:40.
51. Burau V, Carstensen K, Fredens M, Kousgaard MB. Exploring drivers and challenges in implementation of health promotion in community mental health services: a qualitative multi-site case study using Normalization Process Theory. BMC Health Serv Res. 2018;18:36.
52. Vinterflod C, Gustafsson M, Mattsson S, Gallego G. Physicians' perspectives on clinical pharmacy services in Northern Sweden: a qualitative study. BMC Health Serv Res. 2018;18:35.
53. Nakrem S, Solbjør M, Pettersen IN, Kleiven HH. Care relationships at stake? Home healthcare professionals' experiences with digital medicine dispensers - a qualitative study. BMC Health Serv Res. 2018;18:26.
54. Mugo NS, Dibley MJ, Damundu EY, Alam A. "The system here isn't on patients' side"- perspectives of women and men on the barriers to accessing and utilizing maternal healthcare services in South Sudan. BMC Health Serv Res. 2018;18:10.
55. Jaeger FN, Bechir M, Harouna M, Moto DD, Utzinger J. Challenges and opportunities for healthcare workers in a rural district of Chad. BMC Health Serv Res. 2018;18:7.
56. Ofoma UR, Dong Y, Gajic O, Pickering BW. A qualitative exploration of the discharge process and factors predisposing to readmissions to the intensive care unit. BMC Health Serv Res. 2018;18:6.
57. Erichsen Andersson A, Frödin M, Dellenborg L, Wallin L, Hök J, Gillespie BM, Wikström E. Iterative co-creation for improved hand hygiene and aseptic techniques in the operating room: experiences from the safe hands study. BMC Health Serv Res. 2018;18:2.
58. Johnson R, Grove A, Clarke A. It's hard to play ball: A qualitative study of knowledge exchange and silo effects in public health. BMC Health Serv Res. 2018;18:1.
59. Grol SM, Molleman GRM, Kuijpers A, van der Sande R, Fransen GAJ, Assendelft WJJ, Schers HJ. The role of the general practitioner in multidisciplinary teams: a qualitative study in elderly care. BMC Fam Pract. 2018;19:40.
60. Kirkegaard P, Edwards A, Nielsen TLO, Ørntoft TF, Sørensen KD, Borre M, Bro F. Perceptions about screening for prostate cancer using genetic lifetime risk assessment: a qualitative study. BMC Fam Pract. 2018;19:32.
61. Sowińska A, Czachowski S. Patients' experiences of living with medically unexplained symptoms (MUS): a qualitative study. BMC Fam Pract. 2018;19:23.
62. Prang KH, Canaway R, Bismark M, Dunt D, Kelaher M. The use of public performance reporting by general practitioners: a study of perceptions and referral behaviours. BMC Fam Pract. 2018;19:29.
63. Quinn C, Denman K, Smithson P, Owens C, Sheaff R, Campbell J, Porter I, Annison J, Byng R. General practitioner contributions to achieving sustained healthcare for offenders: a qualitative study. BMC Fam Pract. 2018;19:22.
64. Müller CA, Fleischmann N, Cavazzini C, Heim S, Seide S, Geister C, Tetzlaff B, Hoell A, Werle J, Weyerer S, Scherer M, Hummers E. Interprofessional collaboration in nursing homes (interprof): development and piloting of measures to improve interprofessional collaboration and communication: a qualitative multicentre study. BMC Fam Pract. 2018;19:14.
65. Björk Brämberg E, Torgerson J, Norman Kjellström A, Welin P, Rusner M. Access to primary and specialized somatic health care for persons with severe mental illness: a qualitative study of perceived barriers and facilitators in Swedish health care. BMC Fam Pract. 2018;19:12.
66. Wildeboer JA, van de Ven ART, de Boer D. Substitution of care for chronic heart failure from the hospital to the general practice: patients' perspectives. BMC Fam Pract. 2018;19:8.
67. Gallacher KI, May CR, Langhorne P, Mair FS. A conceptual model of treatment burden and patient capacity in stroke. BMC Fam Pract. 2018;19:9.
68. Uhl MC, Muth C, Gerlach FM, Schoch GG, Müller BS. Patient-perceived barriers and facilitators to the implementation of a medication review in primary care: a qualitative thematic analysis. BMC Fam Pract. 2018;19:3.
69. Stark A, Kaduszkiewicz H, Stein J, Maier W, Heser K, Weyerer S, Werle J, Wiese B, Mamone S, König HH, Bock JO, Riedel-Heller SG, Scherer M. A qualitative study on older primary care patients' perspectives on depression and its treatments - potential barriers to and opportunities for managing depression. BMC Fam Pract. 2018;19:2.
70. Ma F, Bai Y, Bai Y, Ma W, Yang X, Li J. Factors influencing training transfer in nursing profession: a qualitative study. BMC Med Educ. 2018;18:44.
71. Francois J, Sisler J, Mowat 3. Peer-assisted debriefing of multisource feedback: an exploratory qualitative study. BMC Med Educ. 2018;18:36.
72. Mogre V, Stevens FCJ, Aryee PA, Amalba A, Scherpbier AJJA. Why nutrition education is inadequate in the medical curriculum: a qualitative study of students' perspectives on barriers and strategies. BMC Med Educ. 2018;18:26.
73. Mahler C, Schwarzbeck V, Mink J, Goetz K. Students´ perception of interprofessional education in the bachelor programme "Interprofessional Health Care" in Heidelberg, Germany: an exploratory case study. BMC Med Educ. 2018;18:19.
74. Burgess A, Roberts C, Sureshkumar P, Mossman K. Multiple mini interview (MMI) for general practice training selection in Australia: interviewers' motivation. BMC Med Educ. 2018;18:21.
75. Chu SY, Lin CW, Lin MJ, Wen CC. Psychosocial issues discovered through reflective group dialogue between medical students. BMC Med Educ. 2018;18:12.
76. Brooker R1, Hu W1, Reath J1, Abbott P2. Medical student experiences in prison health services and social cognitive career choice: a qualitative study. BMC Med Educ. 2018;18:3.
77. Deldar K, Froutan R, Ebadi A. Challenges faced by nurses in using pain assessment scale in patients unable to communicate: a qualitative study BMC Nurs 2018;17:11.
78. Moquin H, Seneviratne C, Venturato L. From apprehension to advocacy: a qualitative study of undergraduate nursing student experience in clinical placement in residential aged care. BMC Nurs. 2018;17:8.
79. Arora A, Manohar N, Bedros D, Hua APD, You SYH, Blight V, Ajwani S, Eastwood J, Bhole S. Lessons learnt in recruiting disadvantaged families to a birth cohort study. BMC Nurs. 2018;17:7.
80. Bailey JM, Hansen V, Wye PM, Wiggers JH, Bartlem KM, Bowman JA. Supporting change in chronic disease risk behaviours for people with a mental illness: a qualitative study of the experiences of family carers. BMC Public Health. 2018;18:416.
81. Dambach P, Jorge MM, Traoré I, Phalkey R, Sawadogo H, Zabré P, Kagoné M, Sié A, Sauerborn R, Becker N, Beiersmann C. A qualitative study of community perception and acceptance of biological larviciding for malaria mosquito control in rural Burkina Faso. BMC Public Health. 2018;18:399.
82. McKinnon I, Finch T. Contextualising health screening risk assessments in police custody suites - qualitative evaluation from the HELP-PC study in London, UK. BMC Public Health. 2018;18:393.
83. James M, Todd C, Scott S, Stratton G, McCoubrey S, Christian D, Halcox J, Audrey S, Ellins E, Anderson S, Copp I, Brophy S. Teenage recommendations to improve physical activity for their age group: a qualitative study. BMC Public Health. 2018;18:372.
84. Brown KM, Elliott SJ, Robertson-Wilson J, Vine MM, Leatherdale ST. Can knowledge exchange support the implementation of a health-promoting schools approach? Perceived outcomes of knowledge exchange in the COMPASS study. BMC Public Health. 2018;18:351.
85. Woodgate RL, Busolo DS. Above chaos, quest, and restitution: narrative experiences of African immigrant youth's settlement in Canada. BMC Public Health. 2018;18:333.
86. Oria PA, Wijnands M, Alaii J, Leeuwis C. Options for sustaining solar-powered mosquito trapping systems on Rusinga Island, Western Kenya: a social dilemma analysis. BMC Public Health. 2018;18:329.
87. Lasebikan VO, Ayinde O, Odunleye M. Assessment of the alcohol consumption among outdoor bar drinkers in Nigeria by qualitative methods. BMC Public Health. 2018;18:318.
88. Dyrstad SM, Kvalø SE, Alstveit M, Skage I. Physically active academic lessons: acceptance, barriers and facilitators for implementation. BMC Public Health. 2018;18:322.
89. Kagoné M, Yé M, Nébié E, Sié A, Müller O, Beiersmann C. Community perception regarding childhood vaccinations and its implications for effectiveness: a qualitative study in rural Burkina Faso. BMC Public Health. 2018;18:324.
90. Mazumder S, Upadhyay RP, Hill Z, Taneja S, Dube B, Kaur J, Shekhar M, Ghosh R, Bisht S, Martines JC, Bahl R, Sommerfelt H, Bhandari N. Kangaroo mother care: using formative research to design an acceptable community intervention. BMC Public Health. 2018;18:307.
91. Donnachie C, Wyke S, Hunt K. Men's reactions to receiving objective feedback on their weight, BMI and other health risk indicators. BMC Public Health. 2018;18:291.
92. Dong KR, Must A, Tang AM, Beckwith CG, Stopka TJ. Competing priorities that rival health in adults on probation in Rhode Island: substance use recovery, employment, housing, and food intake. BMC Public Health. 2018;18:289.
93. Lee SY, Lee EE. Cancer screening in Koreans: a focus group approach. BMC Public Health. 2018;18:254.
94. Ahorlu CSK, Koka E, Adu-Amankwah S, Otchere J, de Souza DK. Community perspectives on persistent transmission of lymphatic filariasis in three hotspot districts in Ghana after 15 rounds of mass drug administration: a qualitative assessment. BMC Public Health. 2018;18:238.
95. Du Plessis LM, McLachlan MH, Drimie SE. What does an enabling environment for infant and young child nutrition look like at implementation level? Perspectives from a multi-stakeholder process in the Breede Valley Sub-District, Western Cape, South Africa. BMC Public Health. 2018;18:240.
96. Littlewood Z, Greenfield S. Parents' knowledge, attitudes and beliefs regarding sun protection in children: a qualitative study. BMC Public Health. 2018;18:207.
97. Sebire SJ, Toumpakari Z, Turner KM, Cooper AR, Page AS, Malpass A, Andrews RC. "I've made this my lifestyle now": a prospective qualitative study of motivation for lifestyle change among people with newly diagnosed type two diabetes mellitus. BMC Public Health. 2018;18:204.
98. Mardi A, Ebadi A, Shahbazi S, Esmaelzade Saeieh S, Behboodi Moghadam Z. Factors influencing the use of contraceptives through the lens of teenage women: a qualitative study in Iran. BMC Public Health. 2018;18:202.
99. Dunkley E, Ashaba S, Burns B, O'Neil K, Sanyu N, Akatukwasa C, Kastner J, Berry NS, Psaros C, Matthews LT, Kaida A. "I beg you…breastfeed the baby, things changed": infant feeding experiences among Ugandan mothers living with HIV in the context of evolving guidelines to prevent postnatal transmission. BMC Public Health. 2018;18:188.
100. Denford S, Lakshman R, Callaghan M, Abraham C. Improving public health evaluation: a qualitative investigation of practitioners' needs. BMC Public Health. 2018;18:190.
101. Goffe L, Penn L, Adams J, Araujo-Soares V, Summerbell CD, Abraham C, White M, Adamson A, Lake AA. The challenges of interventions to promote healthier food in independent takeaways in England: qualitative study of intervention deliverers' views. BMC Public Health. 2018;18:184.
102. Mette J, Velasco Garrido M, Harth V, Preisser AM, Mache S. Healthy offshore workforce? A qualitative study on offshore wind employees' occupational strain, health, and coping. BMC Public Health. 2018;18:172.
103. Wilford A, Phakathi S, Haskins L, Jama NA, Mntambo N, Horwood C. Exploring the care provided to mothers and children by community health workers in South Africa: missed opportunities to provide comprehensive care. BMC Public Health. 2018;18:171.
104. Thapa S, Hannes K, Buve A, Bhattarai S, Mathei C. Theorizing the complexity of HIV disclosure in vulnerable populations: a grounded theory study. BMC Public Health. 2018;18:162.
105. Bruce Baskerville N, Wong K, Shuh A, Abramowicz A, Dash D, Esmail A, Kennedy R. A qualitative study of tobacco interventions for LGBTQ+ youth and young adults: overarching themes and key learnings. BMC Public Health. 2018;18:155.
106. Njelesani J, Hashemi G, Cameron C, Cameron D, Richard D, Parnes P. From the day they are born: a qualitative study exploring violence against children with disabilities in West Africa. BMC Public Health. 2018;18:153.
107. Gupta H, Lam T, Pettigrew S, Tait RJ. Alcohol marketing on YouTube: exploratory analysis of content adaptation to enhance user engagement in different national contexts. BMC Public Health. 2018;18:141.
